# Supplementary material for: BMI at Discharge from Treatment Predicts Relapse in Anorexia Nervosa: A Systematic Scoping Review
Source: J Pers Med. 2022 May 20;12(5):836. doi: 10.3390/jpm12050836 (PMC9144864; doi:10.3390/jpm12050836)
Supplement: Supplementary file 1 [file jpm-12-00836-s001.zip › jpm-1640625-supplementary.pdf]

**Supplementary Materials**

for

# BMI at Discharge from Treatment Predicts Relapse in Anorexia Nervosa: A Systematic Scoping Review

Stein Frostad, Natalia Rozakou-Soumalia, Ştefana Dârvariu, Bahareh Foruzesh, Helia Azkia,

Malina Ploug Larsen, Ehsan Rowshandel and Jan Magnus Sjögren

*Table S1: Search strategy used in the systematic review*

| Database | Query | Results |
|---|---|---|
| Pubmed | ("anorexi*" OR "anorexia nervosa") AND (“weight gain” OR “weight restoration” OR “weight increase” OR “weight normalization” OR “weight normalisation” OR “BMI increase” OR “BMI restoration” OR “BMI normalization” OR “BMI normalisation” OR “treatment”) AND (“prognosis” OR “prognostic factor*” OR “predict* factor*” OR “predictor” OR “time to relapse” OR “relapse” OR “recurrence”) | 1700 |
| Cochrane | anorexi* OR anorexia nervosa AND weight gain OR weight restoration OR weight increase OR weight normalization OR weight normalisation OR BMI increase OR BMI restoration OR BMI normalization OR BMI normalisation OR treatment AND prognosis OR prognostic factor* OR predict* factor* OR predictor OR time to relapse OR relapse OR recurrence | 447 |
| PsycNET | "anorexi*" OR "anorexia nervosa" AND “weight gain” OR “weight restoration” OR “weight increase” OR “weight normalization” OR “weight normalisation” OR “BMI increase” OR “BMI restoration” OR “BMI normalization” OR “BMI normalisation” OR “treatment” AND “prognosis” OR “prognostic factor*” OR “predict* factor*” OR “predictor” OR “time to relapse” OR “relapse” OR “recurrence” | 820 |
| Embase (Ovid) | (anorexi* or anorexia nervosa) and (weight gain or weight restoration or weight increase or weight normalization or weight normalisation or BMI increase or BMI restoration or BMI normalization or BMI normalisation or treatment) and (prognosis or prognostic factor* or predictive factor* or prediction factor* or predictor or time to relapse or relapse or recurrence) | 8540 |

*Table S2. Extraction of data relevant for describing relapse predictors and time to relapse in AN from the 19 included studies.*

| Authors & year of publication | DOI | Study design | Participant number (n) & diagnosis | Participant age & gender | Weight restoration treatment [yes (y) /no (n)] | Intervention & follow-up duration | Potential predictors of relapse | Biological predictors of relapse | Psychological predictors of relapse | ED related predictors of relapse | Time to relapse (may include hospitalization) | Conclusions | Limitations |
|---|---|---|---|---|---|---|---|---|---|---|---|---|---|
| **Bodell & Mayer, 2011** | 10.1002/eat.20801 | Observational-Prospective | n=21 (AN) | M (SD) = 26.6 (5.5)<br><br>100% Females | n | Structured behavioural program (weight and eating behavior normalisation)<br><br>FU: 6-9 months | -Percent adipose tissue: whole body MRI | -Percent adipose tissue | NA | NA | NA | -Lower percent adipose tissue after short-term weight normalization is associated with poor clinical outcome in the year following inpatient treatment. | -Results of a secondary analysis of data<br>-Small sample size<br>-MRI used to measure adipose tissue |
| **Carter et al., 2004** | 10.1017/S0033291703001168 | Observational-Prospective | n=51 (AN) | M (SD) = 26.9 (9)<br><br>100% Females | y | Intensive group therapy programme (weight and eating behaviour normalisation)<br><br>FU: 15months | -History of suicide attempts<br>-Previous specialized treatment for ED<br>-Severity of obsessive-compulsive symptoms: PI<br>-Residual concern about shape and weight after discharge: EDE<br>-Excessive exercise immediately after discharge: semi-structured interview<br>-AN subtype<br>-Age of onset of ED<br>-BMI at admission | NA | -History of suicide attempts<br>-Previous specialized treatment for ED<br>-Severity of obsessive-compulsive symptoms<br>-Residual concern about shape and weight after discharge | -Excessive exercise immediately after discharge | M (SD)= 17 (4.1) months | - There is a significant risk of relapse among AN patients who remain well for the first year post-discharge.<br>-Several variables were considered predictors of relapse. | -Retrospective rather than prospective study<br>-Sample size was relatively small<br>-Large number of censored cases<br>-Variability in the time to follow-up |

| | | | | | | | | | | | | | |
|---|---|---|---|---|---|---|---|---|---|---|---|---|---|
| **Carter et al., 2012** | http://dx.doi.org/10.1016/j.psychres.2012.04.037 | Observational-Prospective | n=100 (AN) | M (SD)= 25.4 (7.7)<br><br>95% Females<br>5% Males | y | Multidisciplinary intervention:<br>-Nutritional rehabilitation<br>-Weight restoration<br>-Eradication of binge eating and purging<br>-Group psychotherapy (CBT, DBT & IPT)<br><br>FU: 1 year | -AN subtype<br>-severity of body checking behavior (pre-treatment): PI<br>-Motivation to recover (during treatment): 3 10-point Likert scale designed for this study<br>-Motivation to recover (post-treatment): 3 10-point Likert scale designed for this study<br>-BMI at admission | NA | NA | -BP-AN subtype<br>-severity of body checking behavior (pre-treatment)<br>-decrease in motivation to recover (during treatment)<br>-lower motivation to recover (post-treatment) | 4-9 months | -Individuals with the BP subtype of AN are particularly susceptible to relapse.<br>-Increasing and maintaining motivation to recover during acute treatment may have an important impact on long-term outcome. | -Motivation for recovery was measured with an instrument that has not yet been validated and consisted of three related constructs |
| **Cooper et al., 2021** | https://doi.org/10.1016/j.eatbeh.2021.101518 | Observational-Prospective | n=146 (AN) | M (SD) = 30.1 (14.39)<br><br>92.5% Females<br>7.5% Males | y | Behavioral meal-based protocol to help patients interrupt unhealthy eating and weight control behaviors<br><br>FU: 6 months | -Normative eating self-efficacy: EDRSQ<br>-Body image self-efficacy: EDRSQ<br>-Drive for thinness: EDI-2<br>-Body satisfaction: EDI-2 | NA | NA | -Normative eating self-efficacy | 6 months | -Normative eating self-efficacy and normalized eating behaviors may represent vital treatment targets for relapse prevention interventions for this high-risk population. | -Risk of responder bias, a common confounder of longitudinal outcome studies of intensive treatment for AN<br>-The use of self-reported weight at follow-up |
| **Dardennes et al., 2021** | 10.1002/erv.2830 | Observational-Prospective | n=26 (AN) | M (SD)= 26.5 (4.3)<br><br>100% Females | y | Behavioural nutritional rehabilitation & weight restoration program<br><br>FU: 2 months | -Leptin levels at discharge<br>-Acyl-ghrelin<br>-Obestatin<br>-Oxytocin<br>-PYY<br>-BDNF | -Low Leptin levels after weight restoration | NA | NA | NA | -Leptin level may be a biomarker of early weight relapse after acute inpatient treatment of AN. | -Non-significant results for some of the predictors |
| **El Ghoch et al., 2016** | 10.1038/ejcn.2015.164; | Observational-Prospective | n=54 (AN) | M (SD) = 25.3 (7.4)<br><br>100% Females | y | CBT-E & early assisted eating<br><br>FU: 1 year | -Total fat percentage: DXA scanner<br>-Trunk fat percentage: DXA scanner<br>-BMI | -Lower BMI at discharge | NA | NA | 1 year | -In short-term weight-restored adult females with AN, BMI, but not body fat percentage or distribution, at inpatient discharge is associated with long-term normal weight maintenance. | -Data were collected in a single inpatient unit applying one treatment programme (lowers external validity)<br>-No objective assessment of physical activity levels or dietary intake during follow-up (could have affected weight and body composition and skewed interpretation of results)<br>-No assessment of body composition at 1-year follow-up |

| | | | | | | | | | | | | | |
|---|---|---|---|---|---|---|---|---|---|---|---|---|---|
| **Focker et al., 2015** | 10.1007/s00787-014-0605-0 | Observational-Prospective | n=161 (AN) | M (SD)= 15.2 (1.5)<br><br>100% Females | y | Day patient treatment after short inpatient care or inpatient treatment<br><br>FU: 1 year | -Premorbid BMI<br>-Duration of illness | -Premorbid BMI | NA | NA | 1 year | -There is a significant association between premorbid BMI percentile and BMI percentile at follow-up. | -The use of measured height at first admission for the calculation of the premorbid BMI<br>-The duration of illness is based on recalled data |
| **Howard et al., 1999** | 10.1176/ajp.156.11.1697 | Observational-Retrospective | n=59 (AN) | M (SD) = 24.8 (8.7)<br><br>100% Females | N | Transferred from inpatient treatment to a day hospital program<br><br>FU: NA | -BMI | -Lower BMI | NA | NA | NA | -Inpatients with anorexia nervosa who have the poor prognostic indicators found in this study are in need of continued inpatient care to avoid immediate relapse and higher cost and longer duration of treatment. | -An individual's eating disorder symptoms were measured with the Eating Attitudes Test and the Eating Disorder Inventory at the time of admission to the in patient unit (Outcome misclassification) |
| **Kaplan et al., 2009** | 10.1017/S003329170800442X. | Observational-Prospective (from RCT) | n=93 (AN) | M (SD) = 23.3 (4.6)<br><br>100% Females | n | Behavioral weight restoration program<br><br>FU: 1 year | -Pre-randomization BMI<br>-Rate of weight loss first 28 days after randomization<br>-AN Subtype | -Lower BMI<br>-Higher rate of weight loss | NA | NA | 6-12 months | -Outcome might be improved by achieving a higher BMI during structured treatment programs and on preventing weight loss immediately following discharge from such programs. | -Results were mainly derived from self-report questionnaires (except for EDE interview)<br>-The treatment program included group and single treatment sessions on demand.<br>-Not a RCT<br>-No assessment of core emotions |
| **Kim et al., 2020** | 10.1002/eat.23407 | Observational-Retrospective | n= 41 (AN) | M (SD)= 25 (5.3)<br><br>100% Females | y | Inpatient treatment (weight restoration, activity restriction and dietary intervention)<br><br>FU: 1 year | -Body composition (percent body fat)<br>-Plasma leptin<br>-Predischarge BMI<br>-Duration of illness<br>-Duration of amenorrhea<br>-Age | -Percent body fat<br>-Fat-adjusted leptin<br>-High log leptin | NA | NA | 1 year | -In summary, in recently weight-recovered women with AN, both higher absolute and body fat-adjusted leptin levels were associated with weight maintenance outcome at 1 year after discharge.<br>-Body composition assessment and posttreatment leptin measurements may be informative in identifying patients at higher risk for relapse, and who might, therefore, benefit from an enhanced, targeted relapse-prevention effort. | -Retrospective study design<br>-Outcome categories were defined with a narrow focus on weight maintenance, but future studies with larger sample sizes could build in additional complexities in the definition of 1-year recovery |

| | | | | | | | | | | | | | |
|---|---|---|---|---|---|---|---|---|---|---|---|---|---|
| **Lock et al., 2013** | 10.1002/eat.22175 | Observational-Retrospective (from 5 RCT) | Total n= 111 (AN),<br>n=83(adolescent AN)<br>n=28 (adult AN) | M (SD)= 20.25 (3.95)<br>Adolescents: M (SD)= 14.5 (1.6)<br>Adults: M (SD)= 26 (6.3)<br><br>100% Females | n | -Adolescent AN: FBT, AFT<br>-Adult AN: CBT, medication and a combination<br><br>FU: 1 year | -BMI<br>-Percent body weight<br>-EDE<br>-OBE and other compensatory behaviors | NA | EDE | NA | NA | -Significant weight gain to a particular criterion by the EOT appears to be the best predictor of recovery for adolescents with AN.<br>-Unfortunately, because so few adults with AN in the study used for this report met even a lower threshold for recovery, it was not possible to ascertain any EOT predictors associated with a broad transdiagnostic conceptualization of recovery in this group. | -Exploratory study utilizing existing databases of participants and conducting a secondary analysis.<br>-Further, sample sizes are generally small and limit both power to detect effects as well as<br>the generalizability of our findings. |
| **Lund et al., 2009** | 10.1002/eat.20634 | Observational-Prospective | n=79 (AN) | M (SD)= 21.6 (7.7)<br><br>100% Females | n | Inpatient treatment (weight restoration and reduction of psychological distress) Involved medical management, psychotherapy, and dietary intervention.<br><br>FU: 1 year | -Admission & discharge BMI<br>-Rate of weight gain<br>-Length of stay<br>-Weight gain | Rate of weight gain | NA | NA | 1 year | -Rate of weight gain was the only restoration parameter that predicted year 1 outcome. | -Potential for unmeasured confounding factors |
| **McCormick et al., 2008** | 10.1002/eat.20549 | Observational-Retrospective (from NRCT) | Total n=18 (AN)<br>n=10 (follow-up data)<br>n=18 (HC) | M (SD)= 25.6 (7.24)<br><br>66.7% Females<br>33.3% Males | n | Inpatient treatment<br><br>FU: 1 year | -ACC volume | Reduced ACC volume | NA | NA | 1 year | -Reduced right dorsal ACC volume during active AN relates to deficits in perceptual organization and conceptual reasoning.<br>-The degree of right dorsal ACC normalization during treatment is related to outcome. | -Only 56% of AN patients had outcome data available at 1 year |
| **McCormick et al., 2009** | 10.1007/BF03327808<br>. | Observational-Retrospective | n=20 (AN) | M (SD)= 27.6 (9.45)<br><br>100% Females | n | Inpatient treatment<br><br>FU: 1 year | -BMI<br>-BDI<br>-Personality (MMPI-2)<br>-Low self-esteem | NA | Low self-esteem | NA | 1 year | -Improved Low Self-Esteem (LSE) from the MMPI-2, from admission to discharge predicted remission at 1-year post-hospitalization.<br>-Pathological levels of neuroticism may represent a vulnerability factor for AN. | -Small sample size<br>-Definition of outcome |

| | | | | | | | | | | | | | |
|---|---|---|---|---|---|---|---|---|---|---|---|---|---|
| **Redgrave et al., 2021** | https://doi.org/10.3389/fpsyt.2021.641861 | Observational-Prospective | Total n = 191 (AN or OSFED)<br>n = 166 (AN)<br>n = 25 (OSFED)<br><br>n= 99 (follow-up data) | M (SD)= 32.55 (12.29)<br><br>100% Females | y<br><br>(behavioral weight restoration) | Behavioral weight restoration program<br><br>FU: 6 months | -Age<br>-Admission and discharge BMI<br>-Diagnostic subtype<br>-Length of inpatient stay<br>-Lifetime nadir BMI<br>-Total weight gained in treatment<br>-Weight suppression<br>-Markers of severity (illness duration, number of previous general, medical, and specialized eating disorder hospitalizations, and scores on the BDI, EDI-2, and Neuroticism subscale of the NEO-FFI) | Discharge BMI | NA | NA | NA | -Rehospitalization rate between discharge and 6-month follow-up was 24%.<br>-The only predictor of BMI=19 kg/m2 at follow-up was discharge BMI.<br>-The likelihood of a BMI=19 kg/m2 at follow-up was 5-fold higher for those with discharge BMI=19 kg/m2. | -The percentage of participants evaluated in follow-up was lower<br>-Weight at follow-up was self-reported<br>-The short-term 6-month follow-up interval means that participants were still potentially within the window during which risk of relapse remains relatively high |
| **Schebendach et al., 2012** | 10.1002/eat.20922. | Observational-Prospective | n=19 (AN)<br>(data from 16 available) | M (SD)= 25.85 (3.85)<br><br>100% Females | y, structured behavioral program aimed at normalizing weight and eating behavior | Multidisciplinary intervention:<br>-Structured behavioral program (normalizing weight & eating behavior)<br>-Individual (supportive ,cognitive & behavioral elements), group and family therapy<br>-Weight restoration<br><br>FU: 1 year | -Total energy<br>-Macronutrient intake<br>-DEDS<br>-DVS | NA | NA | NA | 9-12 months | -A lower DEDS, and possibly a lower DVS, may be associated with poor outcome in recently weight-restored women with AN.<br>-No association was identified between diet variety and treatment outcome. | -This study was a secondary analysis of data<br>-The small sample size clearly increased the risk of a Type II error<br>-Food records are subjective, and the potential effects of misreporting food intake on energy density values should be considered<br>-There is no universally accepted definition of recovery or of relapse in AN |
| **Schulte-Ruther et al., 2012** | https://doi.org/10.1016/j.jaac.2012.06.007 | NRCT | n= 19 (AN)<br>n= 21 (HC) | M (SD)= 15.7 (1.5)<br><br>100% Females | y | Multimodal treatment program:<br>-Nutritional rehabilitation<br>-Weight management<br>-Cognitive-behavioral therapy on an individual and group basis, and family-<br>based interventions.<br><br>FU: 1 year | -Activation of ToM networks:<br>medial prefrontal cortex (mPFC), temporoparietal junction (TPJ), superior temporal sulcus (STS), and temporal pole (TP) | ToM networks | NA | NA | NA | -Brain activation was positively correlated with Morgan-Russell scores, suggesting that reduced ToM-related brain activation at T1 is related to worse outcome<br>-Correlation was not dependent on Grey Matter volume reduction at admission<br>-Impairments of social functioning in anorexia nervosa which is predictive for a poor outcome at 1-year follow-up. | -Group level fMRI does not allow the detection of subtle treatment-related effects.<br>-The sample size was relatively small |

| | | | | | | | | | | | | | |
|---|---|---|---|---|---|---|---|---|---|---|---|---|---|
| | | | | | | | | | | | | -Dysfunction of the mPFC may play a key role for the course of the disorder. | |
| **Seitz et al., 2016** | 10.1007/s00787-016-0819-4 | Observational-Prospective (from RCT) | n= 121 (AN) | M (SD) =15.6 (1.5)<br><br>100% Females | y | Stepped care program of stabilizing inpatient treatment<br>Randomized to inpatient or day patient care<br><br>FU: 1 year | -Leptin levels at discharge<br>-Average rate of weight gain<br>-Duration of illness<br>-Age | Leptin levels Average rate of weight gain | NA | NA | NA | -Leptin levels at discharge and average rate of weight gain were not predictive of weight at 1-year follow-up after referral.<br>-Leptin levels at discharge and the average rate of weight gain during therapy were not significantly correlated. | -The variable for the average rate of weight gain cannot separate patients with a high initial weight gain from those who experience a more substantial weight gain in a later stage of treatment<br>-Did not measure physical activity levels in patients, which are known to be associated with leptin levels in AN |
| **Uniacke et al., 2020** | 10.1002/eat.23269 | Observational-Retrospective (from RCT) | n=93 (AN) | M (SD) = 23.3 (4.6)<br><br>100% Females | y | Weight restoration program<br>CBT and randomized to fluoxetine or placebo<br><br>FU: 1 year | -BMI<br>-Weight suppression | BMI, Weight suppression (WS) | NA | NA | NA | -Weight suppression has no impact on the likelihood of successful weight maintenance or time to relapse.<br>-BMI at the time of study entry was significantly associated with the odds of successful weight maintenance at 6 and 12 months.<br>-There were no significant associations between WS or the odds of successful weight maintenance at 6 or 12 months. Similarly, WS was not significantly associated with time to relapse.<br><br>-The WS × BMI interaction was not significantly associated with successful weight maintenance at 6 or 12 months or with time to relapse | -Secondary analysis<br>-Other metrics of WS may be more useful |

***Abbreviations: ACC: Anterior Cingulate Cortex, AN: Anorexia Nervosa, AFT: Adolescent-Focused Therapy, BDI: Beck Depression Inventory, BDNF: Brain-derived Neurotrophic Factor, BMI: Body Mass Index, BP-AN: Binge/Purge Anorexia Nervosa, CBT: Cognitive Behavioral Therapy, CBT-E: Enhanced Cognitive Behavioral Therapy, DBT: Dialectic Behavioral Therapy, DEDS: Diet Energy Density Score, DVS: Diet Variety Score, DXA: Dual-energy X-ray Absorptiometry, ED: Eating Disorder(s), EDE: Eating Disorder Examination, EDI: Eating Disorder Inventory, EDRSQ: Eating Disorder Recovery Self-Efficacy Questionnaire, EOT: End Of Treatment, FBT: Family-Based Therapy, FU: Follow-up, HC: Healthy Control(s), IPT: Interpersonal Therapy, M (SD): Mean (Standard Deviation), MMPI: Minnesota Multiphasic Personality Inventory, MRI: Magnetic Resonance Imaging, NA: Not Applicable/Available, NRCT: Non-Randomized Control Trial, OBE: Objective Binge Episodes, OSFED: Other Specified Feeding or Eating Disorder(s), PI: Padua Inventory, RCT: Randomized Control Trial.***

*Table S3a. Assessment of risk of bias in observational prognostic studies using the QUIPS tool, domain 1 (Study participation) & domain 2 (Study attrition). Each question/statement was given a "yes" or "no" answer and the summary column indicates the overall bias level for individual domains. These were ranked as low, moderate or high.*

| **Study** | **1. Study participation** **Goal:** *to judge the risk of selection bias s (likelihood that relationship between PF and outcome is different for participants and eligible non-participants)* | | | | | | **2. Study attrition** **Goal:** *To judge the risk of attrition bias (likelihood that relationship between PF and outcome are different for completing and non-completing participants* | | | | | |
|---|---|---|---|---|---|---|---|---|---|---|---|---|
| | Source of target population | Method used to identify population | Inclusion and exclusion criteria | Adequate study participation | Baseline characteristics | **Summary (high, moderate or low risk?)** | Proportion of baseline sample available for analysis | Attempts to collect information on participants who dropped out | Reasons and potential impact of subjects lost to follow up | Outcome and prognostic factor information on those lost to follow up | | **Summary (high, moderate or low risk?)** |
| | The source population or population of interest is adequately described for key characteristics | The sampling frame and recruitment are adequately described, possibly including methods to identify the sample, place and period of recruitment | Inclusion and exclusion criteria are adequately described | There is adequate participation in the study by eligible individuals | The baseline study sample is adequately described for key characteristics | | Response rate is adequate and is > 80% | Is it described? | Is it described? | Participants lost to follow up are adequately described for key characteristics | There are no important differences between key characteristics and outcomes in participants who completed the study and those who did not | |
| Bodell & Mayer, 2011 | Yes | Yes | No | Yes | Yes | Low | Yes | Yes | Yes | No | Yes | Moderate |
| Carter et al., 2004 | Yes | Yes | No | Yes | Yes | Low | Yes | Yes | Yes | Yes | Yes | Low |
| Carter et al., 2012 | Yes | Yes | No | Yes | Yes | Low | Yes | No | No | No | Yes | High |
| Cooper et al., 2004 | Yes | Yes | No | Yes | Yes | Low | Yes | Yes | Yes | Yes | Yes | Low |
| Dardennes et al., 2021 | Yes | Yes | Yes | Yes | Yes | Low | Yes | Yes | Yes | Yes | Yes | Low |
| El Ghoch et al., 2016 | Yes | Yes | Yes | Yes | Yes | Low | Yes | Yes | Yes | Yes | Yes | Low |
| Focker et al., 2015 | Yes | Yes | No | Yes | Yes | Low | Yes | No | Yes | No | No | Moderate |
| Howard et al., 1999 | Yes | Yes | Yes | Yes | Yes | Low | Yes | Yes | Yes | Yes | Yes | Low |
| Kaplan et al., 2009 | Yes | Yes | No | Yes | Yes | Low | No | No | Yes | No | Yes | High |
| Kim et al., 2021 | Yes | Yes | Yes | Yes | Yes | Low | Yes | Yes | Yes | Yes | Yes | Low |
| Lock et al., 2013 | Yes | Yes | Yes | Yes | Yes | Low | Yes | Yes | Yes | Yes | Yes | Low |
| Lund et al., 2009 | Yes | Yes | Yes | Yes | Yes | Low | Yes | Yes | Yes | Yes | No | Moderate |
| McCormick et al., 2008 | Yes | Yes | No | No | Yes | Moderate | No | No | No | No | No | High |
| McCormick et al., 2009 | Yes | Yes | Yes | Yes | Yes | Low | No | No | Yes | No | Yes | Moderate |
| Redgrave et ak., 2021 | Yes | Yes | Yes | No | Yes | Moderate | Yes | Yes | Yes | Yes | Yes | Low |
| Schebendach et al., 2012 | Yes | Yes | No | No | Yes | Moderate | Yes | No | Yes | No | No | Moderate |
| Seitz et al., 2016 | Yes | Yes | No | Yes | Yes | Moderate | No | No | No | No | Yes | High |
| Uniacke et al., 2020 | No | Yes | No | Yes | No | Moderate | No | Yes | No | No | No | High |

*Table S3b. Assessment of risk of bias in observational prognostic studies using the QUIPS tool, domain 3 (Prognostic factor (PF) measurement) & domain 4 (Outcome measurement). Each question/statement was given a "yes" or "no" answer and the summary column indicates the overall bias level for individual domains. These were ranked as low, moderate or high.*

| ***Study*** | **3. Prognostic factor measurement**<br>**Goal:** *to judge the risk of measurement bias related to how PF was measured (differential measurement of PF related to the level of outcome)* | | | | | | | **4. Outcome measurement**<br>**Goal:** *to judge the risk of bias related to the measurement of outcome (differential measurement of outcome related to the baseline level of PF)* | | | |
|---|---|---|---|---|---|---|---|---|---|---|---|
| | Definition of the PF | Valid and Reliable Measurement of PF | | Method and Setting of PF Measurement | Proportion of data on PF available for analysis | Method used for missing data | **Summary (high, moderate or low risk?)** | Definition of the Outcome | Valid and Reliable Measurement of Outcome | Method and Setting of Outcome Measurement | **Summary (high, moderate or low risk?)** |
| | A clear definition or description of 'PF' is provided | Method of PF measurement is adequately valid and reliable to limit misclassification bias | Continuous variables are reported or appropriate cut-points (i.e., not data-dependent) are used | The method and setting of measurement of PF is the same for all study participants. | Adequate proportion of the study sample has complete data for PF variable | Appropriate methods of imputation are used for missing 'PF' data (Intent-to-treat analysis, etc) | | A clear definition of outcome is provided, including duration of follow-up and level and extent of the outcome construct | The method of outcome measurement used is adequately valid and reliable to limit misclassification bias | The method and setting of outcome measurement is the same for all study participants | |
| Bodell & Mayer, 2011 | Yes | Yes | Yes | Yes | Yes | No | Low | Yes | Yes | Yes | Low |
| Carter et al., 2004 | Yes | Yes | Yes | Yes | Yes | Yes | Low | Yes | Yes | Yes | Low |
| Carter et al., 2012 | Yes | Yes | Yes | Yes | Yes | Yes | Low | Yes | Yes | Yes | Low |
| Cooper et al., 2004 | Yes | Yes | Yes | Yes | Yes | Yes | Low | Yes | No | Yes | Moderate |
| Dardennes et al., 2021 | Yes | Yes | Yes | Yes | Yes | Yes | Low | Yes | Yes | Yes | Low |
| El Ghoch et al., 2016 | Yes | Yes | No | Yes | Yes | No | Moderate | Yes | Yes | Yes | Low |
| Focker et al., 2015 | Yes | No | Yes | Yes | Yes | Yes | Low | Yes | Yes | Yes | Low |
| Howard et al., 1999 | Yes | Yes | Yes | Yes | Yes | No | Moderate | Yes | Yes | Yes | Low |
| Kaplan et al., 2009 | Yes | Yes | Yes | Yes | Yes | No | Low | Yes | Yes | Yes | Low |
| Kim et al., 2021 | Yes | Yes | Yes | Yes | Yes | Yes | Low | Yes | Yes | Yes | Low |
| Lock et al., 2013 | Yes | Yes | Yes | Yes | Yes | No | Moderate | Yes | Yes | Yes | Low |
| Lund et al., 2009 | Yes | Yes | Yes | Yes | No | No | Moderate | No | No | No | High |
| McCormick et al., 2008 | Yes | Yes | No | Yes | Yes | No | Moderate | Yes | Yes | Yes | Low |
| McCormick et al., 2009 | Yes | Yes | Yes | Yes | Yes | No | Low | Yes | Yes | Yes | Low |
| Redgrave et ak., 2021 | Yes | No | Yes | Yes | No | No | Moderate | Yes | No | Yes | Moderate |
| Schebendach et al., 2012 | Yes | Yes | Yes | Yes | Yes | No | Low | No | Yes | Yes | Low |
| Seitz et al., 2016 | Yes | Yes | Yes | Yes | No | No | Moderate | No | Yes | Yes | Low |
| Uniacke et al., 2020 | Yes | Yes | No | Yes | Yes | No | Moderate | Yes | Yes | Yes | Low |

*Table S3c. Assessment of risk of bias in observational prognostic studies using the QUIPS tool, domain 5 (Study confounding) & domain 6 (Statistical analysis and reporting). Each question/statement was given a "yes" or "no" answer and the summary column indicates the overall bias level for individual domains. These were ranked as low, moderate or high. The overall risk of bias rating was also low, moderate or high, based on the ranking given to each domain.*

| ***Study*** | **5. Study confounding** **Goal**: *to judge the risk of bias due to confounding (i.e. the effect of PF is distorted by another factor that is related to PF and outcome)* | | | | | | **6. Statistical analysis and reporting** **Goal:** *to judge the risk of bias related to the statistical analysis and presentation of results* | | | | **Overall risk of bias** |
|---|---|---|---|---|---|---|---|---|---|---|
| | Important Confounders Measured | Definition of the confounding factor | Valid and Reliable Measurement of Confounders | Method and Setting of Confounding Measurement | Appropriate accounting for confounding | **Summary (high, moderate or low risk?)** | Presentation of analytical strategy | Model development strategy | Reporting of results | **Summary (high, moderate or low risk?)** | |
| | All important confounders, including treatments are measured | Clear definitions of the important confounders measured are provided | Measurement of all important confounders is adequately valid and reliable | The method and setting of confounding measurement are the same for all study participants. | Important potential confounders are accounted for in the study design and/or analysis | | There is sufficient presentation of data to assess the adequacy of the analysis | The selected statistical model is adequate for the design of the study | There is no selective reporting of results | | |
| Bodell & Mayer, 2011 | No | Yes | Yes | Yes | No | Moderate | Yes | Yes | Yes | Low | Low |
| Carter et al., 2004 | No | Yes | No | Yes | Yes | Moderate | Yes | No | Yes | Moderate | Moderate |
| Carter et al., 2012 | No | Yes | No | Yes | Yes | Moderate | Yes | Yes | Yes | Low | Moderate |
| Cooper et al., 2004 | No | Yes | Yes | Yes | Yes | Low | Yes | No | Yes | Low | Low |
| Dardennes et al., 2021 | Yes | Yes | Yes | Yes | No | Low | Yes | Yes | Yes | Low | Low |
| El Ghoch et al., 2016 | No | Yes | No | Yes | No | High | No | No | Yes | High | High |
| Focker et al., 2015 | No | Yes | Yes | Yes | Yes | Moderate | Yes | Yes | Yes | Low | Low |
| Howard et al., 1999 | Yes | Yes | Yes | Yes | No | Moderate | Yes | No | Yes | Moderate | Moderate |
| Kaplan et al., 2009 | No | No | Yes | Yes | Yes | Moderate | Yes | Yes | Yes | Low | Moderate |
| Kim et al., 2021 | Yes | Yes | Yes | Yes | Yes | Low | Yes | Yes | Yes | Low | Low |
| Lock et al., 2013 | Yes | Yes | Yes | Yes | No | Moderate | Yes | Yes | Yes | Low | Moderate |
| Lund et al., 2009 | Yes | No | Yes | Yes | Yes | Low | Yes | Yes | Yes | Low | Moderate |
| McCormick et al., 2008 | Yes | No | Yes | Yes | No | Moderate | Yes | Yes | Yes | Low | Moderate |
| McCormick et al., 2009 | Yes | No | Yes | Yes | Yes | Low | Yes | Yes | Yes | Low | Low |
| Redgrave et ak., 2021 | No | No | No | Yes | No | High | No | Yes | No | Moderate | Moderate |
| Schebendach et al., 2012 | Yes | No | Yes | Yes | Yes | Low | Yes | Yes | Yes | Low | Low |
| Seitz et al., 2016 | No | No | Yes | Yes | Yes | High | Yes | Yes | Yes | Low | Moderate |
| Uniacke et al., 2020 | No | No | No | No | No | High | Yes | Yes | Yes | Low | High |

*Table S4a: Assessment of risk of bias in a NRCT study using the ROBINS-I tool, bias due to confounding (1.). Each question was answered with a Yes (Y), Probably Yes (PY), No (N), Probably Not (PN) or Not applicable/available (NA). Based on these, the risk of bias judgement was rated as low, moderate or high in each domain.*

| **Study** | **1. Bias due to confounding** | | | **Questions relating to baseline confounding** | | | **Questions relating to baseline and time-varying confounding** | | Risk of bias judgement for bias due to confounding |
|---|---|---|---|---|---|---|---|---|---|
| | 1.1 Is there potential for confounding of the effect of intervention in this study? | 1.2. Was the analysis based on splitting participants' follow up time according to intervention received? | 1.3. Were intervention discontinuations or switches likely to be related to factors that are prognostic for the outcome? | 1.4. Did the authors use an appropriate analysis method (stratification, regression, matching, standardization and inverse probability weighing) that controlled for all the important confounding domains? | 1.5. Were confounding domains that were controlled for measured validly and reliably by the variables available in this study? | 1.6. Did the authors control for any post-intervention variables that could have been affected by the intervention? | 1.7. Did the authors use an appropriate analysis method that controlled for all the important confounding domains and for time-varying confounding? | 1.8. Were confounding domains that were controlled for measured validly and reliably by the variables available in this study? | |
| Schulte-Ruther et al., 2012 | Y | N | NA | PY | PY | PY | PY | Y | Moderate |

*Table S4b. Assessment of risk of bias in a NRCT study using the ROBINS-I tool, bias in selection of study participants (2.) and classifications of interventions (3.). Each question was answered with a Yes (Y), Probably Yes (PY), No (N), Probably Not (PN) or Not applicable/available (NA). Based on these, the risk of bias judgement was rated as low, moderate or high in each domain.*

| **Study** | **2. Bias in selection of participants into the study** | | | | | Risk of bias judgement due to selection of participants | **3. Bias in classification of interventions** | | | Risk of bias judgement due to classification of [illegible] |
|---|---|---|---|---|---|---|---|---|---|---|
| | 2.1. Was selection of participants into the study (or into the analysis) based on participant characteristics observed after the start of intervention? | 2.2: Were the post-intervention variables that influenced selection likely to be associated with intervention? | 2.3 Were the post-intervention variables that influenced selection likely to be influenced by the outcome or a cause of the outcome? | 2.4. Do start of follow-up and start of intervention coincide for most participants? | 2.5. If Y/PY to 2.2 and 2.3, or N/PN to 2.4: Were adjustment techniques used that are likely to correct for the presence of selection biases? | | 3.1 Were intervention groups clearly defined? | 3.2 Was the information used to define intervention groups recorded at the start of the intervention? | 3.3 Could classification of intervention status have been affected by knowledge of the outcome or risk of the outcome? | |
| Schulte-Ruther et al., 2012 | N | NA | NA | PN | N | Moderate | Y | Y | N | Low |

*Table S4c. Assessment of risk of bias in a NRCT study using the ROBINS-I tool, bias due to deviations from intended interventions (4.) and missing data (5.). Each question was answered with a Yes (Y), Probably Yes (PY), No (N), Probably Not (PN) or Not applicable/available (NA). Based on these, the risk of bias judgement was rated as low, moderate or high in each domain.*

| **Study** | **4. Bias due to deviations from intended interventions** | | | | | | Risk of bias judgement due to deviations from intended interventions | **5. Bias due to missing data** | | | | | Risk of bias judgement due to missing data |
|---|---|---|---|---|---|---|---|---|---|---|---|---|---|
| | 4.1. Were there deviations from the intended intervention beyond what would be expected in usual practice? | 4.2 Were these deviations from intended intervention unbalanced between groups and likely to have affected the outcome? | 4.3. Were important co-interventions balanced across intervention groups? | 4.4. Was the intervention implemented successfully for most participants? | 4.5. Did study participants adhere to the assigned intervention regimen? | 4.6. If N/PN to 4.3, 4.4 or 4.5: Was an appropriate analysis used to estimate the effect of starting and adhering to the intervention? | | 5.1 Were outcome data available for all, or nearly all, participants? | 5.2 Were participants excluded due to missing data on intervention status? | 5.3 Were participants excluded due to missing data on other variables needed for the analysis? | 5.4 If PN/N to 5.1, or Y/PY to 5.2 or 5.3: Are the proportion of participants and reasons for missing data similar across interventions? | 5.5 If PN/N to 5.1, or Y/PY to 5.2 or 5.3: Is there evidence that results were robust to the presence of missing data? | |
| Schulte-Ruther et al., 2012 | N | NA | PY | Y | PY | NA | Low | Y | N | Y | PY | Y | Low |

*Table S4d. Assessment of risk of bias in a NRCT study using the ROBINS-I tool, bias in measurement of outcomes (6.) and selection of the reported result (7.). Each question was answered with a Yes (Y), Probably Yes (PY), No (N), Probably Not (PN) or Not applicable/available (NA). Based on these, the risk of bias judgement was rated as low, moderate or high in each domain. The summary indicates the total level of risk of bias of the NRCT study.*

| **Study** | **6. Bias in measurement of outcomes** | | | | Risk of bias judgement due to bias in measurement of outcomes | **7. Bias in selection of the reported result:** *Is the reported effect estimate likely to be selected on the basis of the results from:* | | | Risk of bias judgement in selection of reported results | Summary |
|---|---|---|---|---|---|---|---|---|---|---|
| | 6.1 Could the outcome measure have been influenced by knowledge of the intervention received? | 6.2 Were outcome assessors aware of the intervention received by study participants? | 6.3 Were the methods of outcome assessment comparable across intervention groups? | 6.4 Were any systematic errors in measurement of the outcome related to intervention received? | | 7.1. ... multiple outcome measurements within the outcome domain? | 7.2 ... multiple analyses of the intervention-outcome relationship? | 7.3 ... different subgroups? | | |
| Schulte-Ruther et al., 2012 | PN | PY | Y | PN | Low | N | N | NA | Low | Low |
